# MR. JHORI PATRA

**Published:** 2009

**Authors:** Arijit Coondoo

**Affiliations:** *Professor of Dermatology, Vivekananda Institute of Medical Science, Kolkata, India. E-mail: acoondoo@gmail.com*

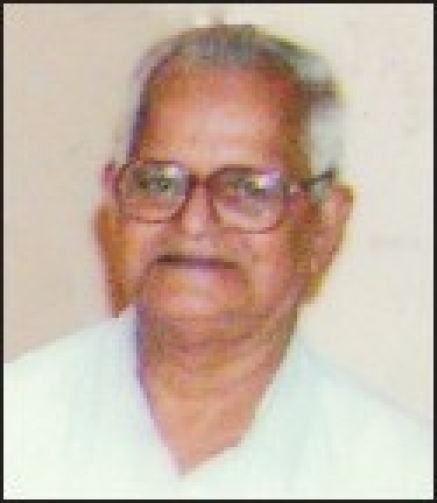



Jhori Patra (alias Jhoru), the heart and soul of the Indian Association of Dermatologists, Venereologists, and Leprologists (IADVL), West Bengal branch, for more than three decades, breathed his last on IADVL day, January 28, 2009.

As a teenager, Jhoru had migrated from his native village in Orissa to war-torn Calcutta in the mid '40s. He was working as an assistant in the chambers of various doctors and also serving as a part-time employee of the Dermatological Society of India in the '60s. With the formation of IADVL he became its full-time employee. In the early days, clad in his dhoti and shirt, he would go all around Calcutta from hospital to hospital, clinic to clinic, on his cycle, distributing meeting notices or the Indian Journal of Dermatology to the members. Even in the extremes of summer, winter or monsoon, Jhoru was untiring in his duty and his smile never left his face.

Later, from 1987, IADVL WB acquired its permanent office and Jhoru was always present in his perch, just inside the main door, ever ready to help in the IADVL work in every possible way, but not before serving tea and biscuits to each IADVL member within a couple of minutes of his entrance.

From the early part of this century Jhoru's age started to tell on him and he kept on requesting us to relieve him of his duties. Conscious about his responsibilities he even brought his replacement Khageshwar from his native place — a teenager whom he trained for a few years before finally retiring in 2005.

Jhoru was a father figure to all of us among the present generation of IADVLites. He was loved by each and every member and was unanimously selected by the Organising Committee to be the man to inaugurate DERMACON 2003. He was quite embarrassed initially — but nevertheless very pleased that he could contribute his tuppence to the cause of IADVL.

Jhoru went back to his native place in Orissa after retirement. But even then IADVL was in his thoughts. He came back once to attend the IJD GOLDCON and CUTICON 2005. Jhoru spent a lot of time advising us, the office-bearers, regarding the future of IADVL WB. One advice was that IADVL WB should procure an apartment to set up a larger office. That was our dream too — and it matured the very next year. Jhoru was very happy when he heard about it. Unfortunately fate intervened before he ever set foot in the new office.

May his soul rest in peace.

